# Gait Difficulty, Postural Instability, and Muscle Weakness Are Associated with Fear of Falling in People with Parkinson's Disease

**DOI:** 10.1155/2012/901721

**Published:** 2011-10-05

**Authors:** Margaret K. Y. Mak, Marco Y. C. Pang, Vincent Mok

**Affiliations:** ^1^Department of Rehabilitation Sciences, The Hong Kong Polytechnic University, Hung Hom, Hong Kong; ^2^Department of Medicine and Therapeutics, Prince of Wales Hospital, The Chinese University of Hong Kong, Hong Kong

## Abstract

The present study aimed to examine the contribution of gait impairment, postural stability and muscle weakness to the level of fear of falling in people with Parkinson's disease (PD). Fifty-seven community-dwelling individuals with PD completed the study. Fear of falling was assessed by the Activities-specific Balance Confidence (ABC) scale. Postural stability and gait difficulty were determined by the posture and gait subscores of the Unified Parkinson's Disease Rating Scale (UPDRS-PG). A Cybex dynamometer was used to measure isokinetic knee muscle strength. Individuals with PD achieved a mean ABC score of 73.6 ± 19.3. In the multiple regression analysis, after accounting for basic demographics, fall history and disease severity, the UPDRS-PG score remained independently associated with the ABC score, accounting for 13.4% of the variance (*P* < 0.001). The addition of knee muscle strength significantly improved the prediction model and accounted for an additional 7.3% of the variance in the ABC score (*P* < 0.05). This is the first study to demonstrate that the UPDRS-PG score and knee muscle strength are important and independent determinants of the level of fear of falling in individuals with PD. Improving balance, gait stability and knee muscle strength could be crucial in promoting balance confidence in the appropriately targeted PD population.

## 1. Introduction

Fear of falling (FoF) is a common and potentially serious problem in people with Parkinson's disease (PD). Previous studies have consistently reported that community-dwelling individuals with PD have a greater FoF than age-matched healthy subjects [1–4]. The level of FoF is further increased in those who have had a fall history [5]. In a prospective study, we found that FoF is also a significant risk factor for predicting future falls [4]. While some level of FoF has a protective role against falls, irrational FoF, either too much or too little, may increase fall risk. Delbaere et al. [6] have recently addressed this complex psychological factor in a large cohort of older population and revealed that discrepancies between psychological and physiological risk factors in those who had excessive or unduly low level of FoF. However, only those with excessive FoF had a higher risk of injurious falls. Repeated falls may lead to avoidance of activity, physical deconditioning, and increased institutionalization. Therefore, interventions aiming to enhance balance confidence have the potential to reduce fall risk in appropriately targeted individuals with PD. 

To design effective treatment intervention, it is crucial to understand the factors that determine FoF. In people with PD, FoF was found to be associated with postural sway in standing and posture and gait impairment as measured by the unified PD rating scale (UPDRS) [1], one-leg stance time, timed-up-and-go time, 6-minute walk distance, and the UPDRS motor score [7]. Jacobs et al. [2] reported that the combination of the pull test, the gait item of the UPDRS, and one-leg-stance time was better than single items in predicting FoF. However, the regression model used in their study did not include factors that could contribute to the prediction of FoF, such as demographic data, disease severity, and fall history. In addition, the association between muscle strength and FoF has not been examined. We recently found that recurrent PD fallers had more lower extremity muscle weakness than PD nonfallers and single fallers [5]. Deficits in muscle power were found to associate with slower gait velocity and increase fall risk in individuals with PD [8]. It is, therefore, important to determine the contribution of muscle strength in predicting FoF. The present study aimed to examine the factors that determine FoF in people with PD. Specifically, we examined balance and gait instability as well as muscle strength, as these are significant fall risk factors in people with PD [3, 9].

## 2. Methods

A convenience sample of 57 individuals with PD completed the study. PD participants were recruited from movement disorders clinics in Hong Kong and the Hong Kong Parkinson's Disease Association, which is a patient self-help group. All patients were diagnosed by neurologists according to the United Kingdom PD Society Brain Bank Criteria [10]. All subjects were recruited on a volunteer basis. Informed consent was obtained from each participant in accordance with the 1964 Declaration of Helsinki, and all experimental work was carried out with the approval of the university ethics committee. To be included in this study, subjects were required to be between 40 and 85 years of age, medically stable, able to walk 6 metres at least three times with and without an assistive device, and able to understand simple commands (minimental state examination score ≥24 [11]). Subjects were excluded if they had neurological conditions other than idiopathic PD, exhibited postural hypotension, visual disturbance, or vestibular dysfunction affecting balance, or had significant cardiovascular or musculoskeletal disorders limiting locomotion or balance. All individuals with PD were tested within 2 hours after medication, that is, during the “on” phase of the medication cycle (Table 1).

## 3. Procedure

All evaluations were carried out at the Hong Kong Polytechnic University gait and motion research laboratory. Demographic data including age, body mass, height, and medications were recorded. We measured disease severity by the Hoehn and Yahr staging scale (HY) [12] and the motor component of the UPDRS [13, 14]. FoF was estimated by the activities-specific balance confidence (ABC) scale [15]. The knee muscle strength of participants was measured by a Cybex Norm dynamometer. Information on the number of fall events over the past 12 months was obtained by patient interview. Participants were classified as fallers if they suffered at least one fall in the past 12 months. A fall is defined as “an event during which a subject comes to rest on the ground or at some lower level, not as the result of a major intrinsic event for example, syncope, stroke and seizure, or overwhelming hazard” [16].

Fear of falling was measured by the validated Chinese version of the ABC scale [17]. Participants were asked to rate their self-perceived balance confidence level from 0 (no confidence at all) to 100 (full confidence) for completing 16 activities of daily living. The mean score was calculated for each subject, with a minimum score of 0 to a maximum of 100. A lower ABC score indicates greater FoF.

The unified PD rating scale motor examination (UPDRS-III) is a valid tool used to assess the level of motor impairment and disability in individuals with PD [13, 14]. It consists of 14 items which assess PD-specific impairments. Each item scores from 0 to 4, with 0 indicating absence of impairment and 4 indicating severe impairment. In this study, the sum of items 27–30 (i.e., rising from a chair, posture, gait, and postural stability (UPDRS-PG) was used to document the postural instability and gait difficulty of PD participants [1]. 

Knee muscle strength was quantitatively assessed by a Cybex Norm isokinetic dynamometer (Lumex, Inc., Ronkonkoma, NY, USA). The more affected lower extremity, which was determined by a higher unilateral UPDRS-III score, was assessed. Participants were seated with their lower leg at 90° of knee flexion, and a strap and a footplate were attached to their lower leg and feet, respectively. Participants were stabilized by trunk and thigh straps during the test. The investigator then measured an anatomical zero when the knee was passively moved to full extension. Participants were instructed to perform isokinetic concentric and eccentric contraction of the knee flexors and extensors from 10° to 70° of flexion at an angular speed of 90°/s. The order of the 4 testing conditions was randomized. Participants were allowed to practice each type of contraction at their submaximal effort 2 times, which was followed by the test trial when 3 maximum concentric or eccentric contractions were performed. Participants were given a 3-minute rest between each mode of contraction. The average value of the peak torque (Nm) among the 3 test trials was obtained, and the sum of mean concentric and eccentric knee muscle strength was used for further analysis. Overall, these strength testing procedures lasted for 20 minutes.

## 4. Statistical Analysis

All statistical analyses were performed using SPSS 17.0, and a significance level of 0.05 (2-tailed) was set for all statistical tests. The Shapiro Wilk statistic was used to check data normality. Descriptive analysis was performed for the demographic data and variables of interest. Bivariate correlation analyses were performed. Pearson product moment correlation was performed to establish the relationship between the ABC score and knee muscle strength, as the data were normally distributed. For the UPDRS-PG score, which is an ordinal data, the relationship with the ABC score was determined by Spearman's rho. A hierarchical multiple linear regression model (enter strategy) was used to determine the contribution of the UPDRS-PG score and knee muscle strength to the ABC score after accounting for other potential contributing factors (e.g., demographic data, fall history, and disease severity measured by the HY staging score). Age, duration of PD, fall history, and the HY staging scores were first entered into the regression model followed by the UPDRS-PG scores and knee muscle strength.

## 5. Results

The mean ABC score for individuals with PD was 73.5 ± 19.3. Individuals with PD had a median HY score of 2.5 ± 1.0, indicating mild-to-moderate disease severity. The median UPDRS-PG score was 4.0 ± 2.0, implying mild gait and postural instability. The mean knee muscle strength was 34.4 ± 13.3 Nm. Correlation analysis showed that the ABC score was positively correlated with knee muscle strength (*r* = 0.301, *P* = 0.029) and inversely correlated with the UPDRS-PG score (*r* = − 0.661, *P* < 0.001). These findings indicate that a higher level of FoF was associated with greater knee muscle weakness and increased gait instability and postural difficulty. The results of the regression model show that after adjusting for basic demographics, fall history, and disease severity, the UPDRS-PG score remained independently associated with the FoF level, accounting for 13.4% of the variance (Model 2, Table 2). The addition of knee muscle strength significantly improved the model prediction by 7.3% (Model 3, Table 2). A total of 47.9% of the variance in the ABC score was predicted by the final regression model (*F*_6,56_ = 6.895, *P* < 0.001). Among all the variables, the UPDRS-PG score was the most important determinant of the ABC score, as reflected by the magnitude of the regression coefficient (*β* = −0.531).

## 6. Discussion

Our PD participants had a mean ABC score of 73.6 ± 19.3, indicating that they had moderate level of FoF. This finding is consistent with the published data [1, 2, 4, 5, 7]. The negative association between FoF and the UPDRS-PG score concurs with previous findings. Excessive FoF was shown to be negatively correlated with the UPDRS-PG score [1, 7], centre of pressure sway during standing [1], and Berg's balance score, tandem Romberg, and timed up and go time [18] in individuals with PD. Our finding extends that reported by Jacobs et al. [2] that postural instability and gait impairment as measured by the UPDRS-PG score is an important determinant of FoF, after accounting for demographic data, fall history, and disease severity in individuals with PD. The UPDRS-PG score alone accounts for 13.4% of the variance of the ABC score. The UPDRS-PG score quantifies participants' standing upright posture, response to retropulsion, sit-to-stand transfer, and gait stability. Stooped posture in people with PD was found to be destabilizing [19] and capable of predicting future falls [9]. In addition, people with PD are known to be slow and inflexible in response to external perturbation, especially to a backward pull [20–22]. Walking and rising from a chair have often been reported to be fall-related activities [23, 24]. For example, 24%–46% of individuals with PD were reported to have fallen during walking and turning and 15% of individuals with PD fell during transferring from sitting to standing [5, 23, 24]. Greater postural instability and gait difficulty in individuals with PD will lead to less perceived self-confidence in performing balance activities, hence an increased level of FoF. 

Previous studies reported that people with PD had reduction in knee and ankle muscle strength [25–27], which was correlated with sit-to-stand performance [28] and gait velocity [26]. Our study is the first to report that knee muscle strength, which accounted for 7.3% of the variance of the ABC score after accounting for demographic data and the UPDRS-PG score, is another important determinant of FoF. In a recent study, we reported that recurrent PD fallers had significantly more reduced lower extremity muscle strength than single fallers [5]. These recurrent fallers also perceived that “muscle gives way” was associated with their falls. Knee muscle strength is crucial for maintaining stability in an upright position. Weakness in this muscle group could give the patients the perception that their “muscles give way” while in the standing position and lead to a lack of confidence in performing standing or walking activities. Lower extremity muscle strength was independently associated with reduced bone mass in individuals with PD [27]. Furthermore, knee extensors muscle strength of the more affected side was a significant fall predictor [9]. When combined with excessive FoF, muscle weakness may restrict individuals' activities, lead to further muscle weakness and accelerated loss of bone mass, and increase the risk of fall-related fracture.

To prevent falls in people with PD, treatment interventions should enhance both physical function and balance confidence. A recent systematic review reported that in older adults, exercise was the most commonly used intervention to improve balance confidence [29]. The exercise interventions include strength, balance, and gait training. Combined cognitive behavioral education (i.e., identification of fall risk factors, discussion of coping strategies for falling, and assertiveness training) and exercise training were found to be effective in enhancing balance confidence and reducing the risk of falls in older people [30]. Based on the significant association between FoF and postural and gait impairment and knee muscle weakness, clinicians may consider incorporating muscle strengthening programmes, as well as improving patients' postural and gait stability, in their fall prevention programs. We believe that the promotion of balance confidence can prevent the vicious cycle of activity restriction, physical deconditioning, further decline in self-perceived balance confidence, and future falls. Further interventional study is needed to prove this postulation.

We acknowledge that our study has certain limitations. To be included in the study, participants needed to be able to walk freely to undertake the gait assessments. Our findings, therefore, are not generalisable to individuals with PD with significant gait impairments. Our assessments were also restricted to “on phase” periods. It is possible that conducting assessments during the “off” phase of treatment would increase their sensitivity. In addition, FoF is associated with many factors. However, we could not include many predicting variables in the regression analysis due to our small sample size. Our model was able to predict 47.9% of the variance of the ABC score. Other physical factors such as freezing of gait and cognitive psychological factors such as cognitive impairment, anxiety, and depression could contribute to the level of FoF. Finally, this is a cross-sectional study. We could not establish a causal relationship between postural impairment, gait difficulties, muscle weakness, and FoF. Further research should address the temporal relationship between postural and gait impairment as well as muscle weakness and FoF. 

To conclude, postural instability, gait difficulty, and knee muscle weakness are important determinants of the level of FoF. The clinical implication of our study is that the balance confidence of people with PD may be enhanced through promoting muscle strength, balance, and gait stability, thereby preventing activity restriction and physical deconditioning and reducing fall risk. Further intervention study is needed to prove this postulation.

## Figures and Tables

**Table 1:** Subject characteristics.

	People with PD (*N* = 57)
*Demographics*	
Age (years)	63.7 (8.5)
Height (cm)	161.2 (8.1)
Weight (kg)	61.1 (10.1)
Female gender, *n* (%)	22 (38.6)
Fallers, *n* (%)	19 (33.3)
*Parkinson's disease characteristics*	
Years since diagnosis of Parkinson's disease (years)	7.6 (4.6)
Hoehn and Yahr stage (0–5)	2.5 (1.0)^#^
UPDRS—motor score III (0–108)	22.6 (6.5)
UPDRS-PG (0–16)	4.0 (2.0)^#^
Knee muscle strength (Nm)	34.4 (13.3)
ABC score (0–100)	73.6 (19.3)

Data shown are means (standard deviations), ^#^median (interquartile range), ABC: activities-specific balance confidence, UPDRS: unified Parkinson's disease rating scale, UPDRS-PG: unified Parkinson's disease rating scale (items 27–30).

**Table 2:** Multiple regression analysis for predicting the ABC score.

Independent variable	*R* ^2^	*R* ^2^ change	*B* (*S.E*.)	*β*	*P* value
*Model 1*	0.272	0.272			
Age			−0.827 (0.307)	−0.359	0.010*
Years since diagnosis			−0.111 (0.553)	−0.028	0.841
Fall history			−5.982 (4.991)	−0.159	0.237
HY stage			−7.243 (5.606)	−0.190	0.203
*Model 2*	0.406	0.134			
Age			−0.636 (0.287)	−0.276	0.032*
Years since diagnosis			0.029 (0.507)	0.007	0.954
Fall history			−4.682 (4.576)	−0.124	0.312
HY stage			0.645 (5.676)	0.017	0.910
UPDRS-PG			−4.630 (1.439)	−0.458	0.002**
*Model 3*	0.479	0.073			
Age			−0.554 (0.274)	−0.240	0.049*
Years since diagnosis			−0.113 (0.484)	-0.028	0.816
Fall history			−2.386 (4.427)	−0.063	0.593
HY stage			2.828 (5.443)	0.074	0.606
UPDRS-PG			−5.371 (1.394)	−0.531	<0.001***
Knee muscle strength			0.040 (0.016)	0.285	0.016*

*P < 0.05, **P < 0.01, ***P < 0.001, *B*: unstandardized regression coefficient, S.E.: standard error, *β*: standardized regression coefficient, ABC: activities-specific balance confidence, UPDRS-PG: unified Parkinson's disease rating scale (items 27–30), HY: Hoehn and Yahr.
